# Emotional regulation strategies in daily life: the intensity of emotions and regulation choice

**DOI:** 10.3389/fpsyg.2023.1218694

**Published:** 2023-08-14

**Authors:** Magdalena Kozubal, Anna Szuster, Adrianna Wielgopolan

**Affiliations:** Faculty of Psychology, University of Warsaw, Warsaw, Poland

**Keywords:** emotion regulation strategies, rumination, reappraisal, acceptance, distraction, suppression, intensity of emotions, daily diary study

## Abstract

**Objective:**

Emotion regulation is an adaptive ability affecting people’s physical and mental health, quality of life and functioning. In the present study we focused on the influence of the intensity of experienced emotions on emotion regulation strategies (ERS) that are applied in everyday life.

**Methods:**

For 7 days the participants kept an online diary where every day they described the situation which had aroused their strongest negative emotions. Next, they identified the emotions, their intensity and the type of applied strategies (acceptance vs. reappraisal vs. rumination vs. distraction vs. suppression). The study involved 88 people *N* = 88, which gives 538 observations.

**Results:**

The obtained results indicate that the intensity of emotions affects the choice of regulation strategies. When the intensity increases, people are more likely to choose the rumination strategy and less likely to choose the reappraisal strategy. However, the expected relationship between the intensity and the number of regulation strategies was not confirmed. In turn, it was gender (male) that turned out to be associated with a greater number of strategies used.

**Conclusion:**

The concern of this research was to look at making regulatory decisions in personally relevant and complex everyday situations. Although the emotions experienced in response to a difficult situation were varied, the intensity of the emotional experience was an important factor determining the choice of a regulation strategy. It indicates that this emotional dimension is a basic and determining aspect in people’s regulatory capabilities. These results also indicate that perhaps men in a situation perceived as stressful and worthy of emotional involvement use more regulatory strategies than women. These findings may find an application in all kinds of psychological interventions (e.g., psychotherapy, anger management therapies).

## 1. Introduction

Emotions are one of the crucial psychophysiological processes which help people adapt to the environment and achieve their goals (Eisenberg, 2000). Rapidly occurring social changes and the development of digital technology, which can be observed in recent years, confront people with unprecedented multitude and diversity of stimuli. It generates cognitive strain, situational stress and carries negative affective states. Over the last 2 years it was the COVID-19 pandemic and concurrent restrictions that were the particular source of a long-term threat. Due to such changes, the ability to flexibly regulate emotional reactions has become something even more valuable than before.

Emotion regulation is connected with physical (Sapolsky, 2007) and mental (Gross and Muñoz, 1995) health. Effective regulation is a factor restraining depression, correlating with work efficiency (Diefendorff et al., 2000), relationships satisfaction (Rick et al., 2017) and commonly perceived wellbeing (Garnefski et al., 2001; Gross and John, 2003). Even though the issue of emotion regulation is a relatively new field of scientific studies, a number of research, mainly of the questionnaire (Garnefski et al., 2001; Gross and John, 2003; Aldao and Nolen-Hoeksema, 2010) or experimental (Butler et al., 2003; Webb et al., 2012; Gross and Thompson, 2014) character, have already been conducted. In the latter type of research the participants were usually instructed to apply one specific regulation strategy in response to an unpleasant stimulus. It allowed making a relative comparison of particular ERS (Webb et al., 2012). However, such a procedure does not reflect real situations. People usually experience a mix of emotions which vary both when it comes to their content and intensity. Also, emotion regulation is a spontaneous process. The newest research results indicate that people who have a free choice usually apply a lot of ERS (Ehring et al., 2010; Aldao and Nolen-Hoeksema, 2012; Szasz et al., 2018). In the research conducted by Opitz et al. (2015), in spite of the instruction telling the participants to employ the reappraisal strategy, between 50 and 75% of them interchangeably or additionally used other strategies. Studies also show that not all strategies are applied with the same frequency (Dixon-Gordon et al., 2015). Scholars are currently paying more attention to the significance of contextual factors which influence the selection and effectiveness of ERS, i.a. because of the need to verify the obtained results by means of more ecological validity methods (Mitchell et al., 2012). As a result, more and more studies are being conducted by the use of the longitudinal, daily diary or momentary report method. Such studies refer to their participants’ everyday experiences. In our study we focused on the influence of one of the most basic dimensions of emotions, namely their intensity, on the selection of a regulation strategy. In order to verify the results of laboratory research in this area, we applied the more ecological daily diary method, according to which the participants described their everyday experiences and their emotional reactions in unpleasant situations.

### 1.1. Emotion regulation strategies

People normally modulate their reactions by means of a wide range of regulation strategies (Gross and Thompson, 2014). Emotion regulation is defined as “the processes by which individuals influence which emotions they have, when they have them, and how they experience and express them” (Gross et al., 1998) (p. 275). The regulation can be introduced at any stage of the emotional process, either before or after the occurrence of the emotional response. It differentiates anticipative strategies and strategies related to the emotional response itself. According to the Gross’s model, there are five ERS groups: situation selection, situation modification, attentional deployment, cognitive change and response modulation (Gross, 2012). The study presented below focused on five regulation strategies: reappraisal, rumination, distraction, suppression and acceptance. The selection was based, *inter alia*, on the results of the meta-analysis of ERS effectiveness (Webb et al., 2012).

The emotional process is initiated with attention deployment which is essentially based on directing attention in order to modify one’s emotional state (Gross and Thompson, 2014). Distraction is the strategy thanks to which an individual focuses their attention on different aspects of a given situation or distracts attention from it. It can also involve a change of one’s inner direction, for instance when people recall thoughts or memories which are in discrepancy with an undesirable emotional state (Gross and Thompson, 2014). Distraction results effective mostly in situations when it is connected with a conscious effort, not merely with deflection caused by other stimuli or a task given by the researchers (Locke and Latham, 1990; Webb et al., 2012). The rumination strategy, which is based on concentration on negative situations, emotions and thoughts triggered by them, has the opposite effect since it results in the sustainment of negative emotions, increasing their intensity and prolonging their duration. It is also connected with increased depressive susceptibility (Morrow and Nolen-Hoeksema, 1990). The distraction strategy is perceived as shifting one’s attention to a positive or neutral thought, or focusing one’s attention on a different activity. The rumination strategy is perceived as concentrating one’s attention on thoughts and feelings concerning an unpleasant situation or analysing its causes and consequences. The most extensive category of mental regulation techniques is the cognitive change. It is defined as a change of thinking about a given situation or the opportunities of handling it (Gross and Thompson, 2014). Just like the attention shift, the cognitive change is one of the anticipative strategies applied when a situation triggering negative emotions cannot be avoided or changed. Mental attitude becomes an effective way to change one’s emotional state then. However, tasks used in research on the cognitive change are extremely varied. They concern different aspects and ways of perceiving an emotional experience. We can distinguish those ones which encouraged reassessing a given situation and detecting its positive consequences, and those which led to adopting another perspective, for instance the observer’s perspective, or reappraising the emotional experience itself (Webb et al., 2012). Some scholars also employed the combination of the above mentioned strategies. We focused on the cognitive change strategy considered as reappraisal, that is assigning a different meaning to a given situation or taking the perspective of a different person.

Nowadays the interactions based on mindfulness, enriched with the diversity of therapeutical approaches, are becoming more and more popular, for example in ACT (Hayes et al., 2012), DBT (Linehan et al., 1994), MBCT (Segal et al., 2018). They put special emphasis on the role of acceptance in the regulation of the emotional process. Applying it in everyday life is connected with lowering the negative affect (Kashdan et al., 2006; Shallcross et al., 2010; Wojnarowska et al., 2020) and physiological reactivity in response to a negative stimulus triggering negative emotions (Troy et al., 2018). Some scholars perceive this strategy as the cognitive change, the reappraisal of the emotional state, since people usually assess their negative emotional condition and do not approve it. In our study we considered the acceptance strategy as acknowledging a given situation, oneself and one’s own emotional state, accepting them the way they were without assessing them.

Suppression is the strategy based on modulating the emotional response which has already occurred, and inhibiting emotion expressing behavior. It can refer merely to supressing emotional expression, but also to feelings or thoughts regarding a given situation. The suppression of emotional expression resulted effective in lowering the intensity of the emotional response. Nonetheless, the suppression of experienced emotions and thoughts resulted to have the opposite effect or even intensify physiological reactions of one’s organism (Gross and John, 2003). The research on applying the suppression strategy revealed that the ability to use both expression and suppression of emotions is important in maintaining mental health. Whereas the frequency of supressing emotions is linked with inefficient adoption, the ability to adopt emotional expression to a given situation brings positive clinical and social results (Bonanno et al., 2004; Gupta and Bonanno, 2011). In the present study suppression is understood as supressing thoughts, feelings and emotional expression.

In the above mentioned experimental studies, the research procedure usually imposed on the participants the choice of a specific strategy. The spontaneous choice of a regulation strategy depends on different internal and external aspects, for instance those connected with individual differences or the situational ones, resulting from the experienced emotion itself.

### 1.2. Intensity of emotions and selection of ERS

Intensity of a given emotion is one of its most characteristic features. It exerts an influence on people’s motivation and behavior. Emotional intensity is reflected in a subjective experience, but also in the form of mimic, visceral and nervous reactions (Lang et al., 1993; Sonnemans and Frijda, 1995). It is a variable feature and often does not reach the maximum level. Just like in the case of the emotion itself, its duration is limited. Variations on the emotional activity scale are regarded to be relatively independent from the type of an experienced emotion, its indication (positive or negative) or content (Diener and Iran-Nejad, 1986; Schimmack and Diener, 1997). What seems to be an important determinant of emotional intensity is the motivational meaning of a given situation and its influence on goals established by an individual (Lazarus, 1991; Sonnemans and Frijda, 1995; Brehm, 1999; Scherer, 1999). Regulation changes in the emotional process occur mainly within intensity (decrease/increase) and temporal features such as the moment the emotion appears or its persistence (Thompson, 1990).

Recent studies indicate that emotional intensity exerts an influence on the selection of applied regulation strategies (Suri et al., 2018). More intensive negative emotions require people to apply a higher number of regulation strategies (Barrett et al., 2000; Gross and Thompson, 2014; Dixon-Gordon et al., 2015). It may result from a higher motivation for change, and consequently the effort put into regulation in case of experiencing an unpleasant emotional state. Studies also point out that desadaptive strategies such as avoiding are more often chosen when emotional intensity is higher (e.g., Sheppes et al., 2011, 2014; Sheppes and Levin, 2013). A series of comparative experimental studies, regarding the selection of either distraction or reappraisal while being presented a stimulus triggering low or high emotion intensity, indicate that people who have a free choice prefer to apply reappraisal when the emotion intensity is low, and distraction when the emotion intensity is high (Sheppes et al., 2011, 2014). The same pattern was observed in relation to various stimuli (negative pictures, electrocuting; Sheppes et al., 2011) and in the study where the participants were offered a financial reward for applying the less preferable strategy (Sheppes et al., 2014). Such results probably arise from a difference in the undertaken cognitive effort aiming at changing the emotional arousal. Distraction, as the strategy based on backing out and lack of engagement, requires less cognitive resources than reappraisal. Studies indicate that while making decisions people tend to minimize cognitive effort (Kool et al., 2010; Tversky and Kahneman, 2018), especially in situations connected with a high level of stress and danger (Muraven and Baumeister, 2000).

There are not many pieces of research which were conducted in natural conditions and concerned the meaning of emotion intensity in applying regulation strategies. Nevertheless, their results indicate that the use of ER strategies is more complex in daily life than in a laboratory. Lennarz et al.’s (2019) study involving the momentary report method, conducted with the participation of a group of adolescents, demonstrated that in case of low intensity negative emotions teenagers more often applied the acceptance strategy, whereas in case of high intensity negative emotions they more frequently employed the suppression, distraction and rumination strategies. There was no significant relationship between reappraisal and emotional intensity in the study. Adult studies indicate that reappraisal is used more often when emotional intensity is lower (Troy et al., 2018; Mehta et al., 2020; Wilms et al., 2020; Blanke et al., 2021) however, in Ortner and Pennekamp (2020) research it was unrelated, whereas rumination is used more often when emotional intensity is higher (Ortner and Pennekamp, 2020; Wilms et al., 2020; Blanke et al., 2021). The use of distraction was associated with a higher intensity of negative emotions compared to reappraisal (Mehta et al., 2020) which was unrelated (Wilms et al., 2020; Blanke et al., 2021) or less frequently used in other studies (Ortner and Pennekamp, 2020). There is not much research on the remaining two strategies. Acceptance was used less frequently when stressors were more intense (Blanke et al., 2021). Expressive suppression was negatively associated with experience intensity (Ortner and Pennekamp, 2020).

Currently available research results imply that emotion intensity influences the selection of ERS. However, the disproportion between laboratory tests and surveys carried out in everyday life on the ERS choice is still very large. The results obtained in ecological conditions are valid compared to those provided by laboratory tests. Therefore, both approaches should be treated as complementary. Furthermore, the already conducted experience sampling studies on the impact of emotions intensity on the choice of regulation strategies differ in the methodology used and the results obtained. This indicates the need for further research in this area and the replication of results by the use of different methodologies.

### 1.3. Aim and hypotheses

The aim of the study was to determine the influence of experienced emotions and their intensity on the selection of ERS. In order to ensure the ecological validity character of the procedure, the daily diary method was employed. For 1 week the participants every day described the situation which triggered their strongest unpleasant emotions. They entered their emotions independently and then assessed their intensity. Therefore, they could fully report their emotional state without the risk that it did not fit into one of the given categories. They also answered the question which regulation strategies they had applied in these situations.

The research results indicate that emotion intensity can influence the selection of specific regulation strategies (Sheppes et al., 2014; Wilms et al., 2020; Blanke et al., 2021). Due to the fact that strong arousal concentrates attention and cognitive resources on the stimulus provoking the emotional response (Mather and Sutherland, 2011; Markovic et al., 2014) we suppose that high emotion intensity will increase the likelihood of selecting the rumination strategy. Strong arousal does not favor the cognitive emotion regulation though (Veilleux et al., 2021). Therefore, we believe that the higher the emotion intensity, the less frequently the reappraisal strategy will be applied. From the cognitive point of view, the indications of the reappraisal strategy are the most varied ones. It is the most extensive category of regulation strategies, the determinant of which is the reappraisal of an emotional experience. The described aspects are therefore connected with various levels of cognitive effort and the complexity of cognitive mechanisms.

In accordance with the foregoing research results (Gross and Thompson, 2014; Dixon-Gordon et al., 2015), we assume that higher intensity of emotions will be associated with the use of more strategies.

Hypothesis 1 (H1). The higher the emotion intensity, the more likely it is for the rumination strategy to be chosen.

Hypothesis 2 (H2). The higher the emotion intensity, the less likely it is for the reappraisal strategy to be chosen.

Hypothesis 3 (H3). The higher the emotion intensity, the more regulation strategies will be employed.

## 2. Materials and methods

### 2.1. Participants

The study involved 180 adults. The participants were recruited by method of snowball sampling. The analysis included the results of those participants who completed the diary at least for five out of 7 days *N* = 88; (aged from 18 to 71; *M* = 36.30; *SD* = 11.66), including 27,3% men (*N* = 24). In total we gathered 538 observations from 88 participants.

### 2.2. Materials

#### 2.2.1. Emotion intensity

The participants assessed the intensity of experienced emotions on the scale from 1 to 10, where 1 stood for *very low* whereas 10 stood for *very high* intensity. The rate of emotion intensity for each and every observation was represented by the average of the intensity of identified emotions.


$$M=\left(e_{1}+e_{2}+\ldots e_{n}\right)/N$$

e_1 – intensity of the first identified emotion,_ e_2 – intensity of the second identified emotion,_ e_n – intensity of the last identified emotion_, *N* – _number of identified emotions_

If a person identified 5 emotions concerning a given situation on a given day, the emotion intensity on this day was calculated as the average of the intensity of these 5 emotions.

#### 2.2.2. Type of strategy

There were 15 randomly ordered sentences describing behaviors which represented the strategies of acceptance, distraction, rumination, reappraisal and suppression. The participants could choose as many sentences as they wanted to. The selection of strategies was based on the meta-analysis of the studies on ERS conducted by Webb et al. (2012). The frequency of applying ERS was measured by means of the following instruction: “Mark if in this situation:” Marking at least one of the sentences representing a specific strategy served as the indicator for applying particular strategies. 0 – no strategy was applied, 1 – applying the strategy (marking one or more descriptions assigned to each strategy). The list of sentences representing 5 emotion regulation strategies is presented in Supplementary Appendix 1.

### 2.3. Procedure

A special website including the diary was created, which enabled the participants to complete the diary every day and describe the situation that aroused their strongest unpleasant emotions. After describing the situation they answered the question what emotions they were feeling during the occurrence. They named the emotions on their own, yet they could also use prompts and look at the specially prepared list of emotions (Supplementary Appendix 2). Next, they assessed the intensity of each experienced emotion on the scale from 1 to 10, illustrated in the form of a slider displayed under the scale. The last measurement concerned the applied ERS. It consisted of 15 questions describing the way of reacting to experienced emotions, identifying particular strategies of reappraisal, acceptance, distraction, suppression and rumination (3 sentences regarding each strategy). The participants’ task was to choose those descriptions that reflected their own way of reacting in response to the experienced situation, for example: *I accepted the feelings which were triggered inside of me by this situation.* The participants could mark as many sentences as they wanted to. If none of the descriptions suited their reacting method, they were asked to describe it in their own words. Nevertheless, such cases happened rarely, and the answers were not coherent enough to be covered by the quantitative analysis. In order to standardize the procedure, the participants were supposed to complete the diary for 7 consecutive days between 5.00 pm and 1.00 am. The people participating in the study had no diagnosed mental disorders and had not experienced any serious stressors in their lives (such as the death of a loved one, loss of a job, moving house, etc.) during or at least 1 month before the study. Each participant could resign from completing the study at any time and had the opportunity to contact a psychologist. The survey was completed on private computers of the respondents.

### 2.4. Data analysis

In order to verify the relationship between intensity of emotions and the type and number of selected ERS in a longitudinal research project we conducted GLMM and LMM analysis. We have created separate multilevel linear regression models for each of five strategies and number of applied ERS. In each model, sex and age were controlled.

## 3. Results

### 3.1. Intensity of emotions and the choice of ERS

We observed that the subjects usually entered more than one emotion. The mean number of reported emotions was 4.56 (SD = 2.86) for the first day, *M* = 3.45, SD = 2.07 for the second, *M* = 3.91, SD = 2.34 for the third, *M* = 3.73, SD = 2.71 for the fourth, *M* = 3.34, SD = 2.48 for the fifth, *M* = 3.68, SD = 2.72 for the sixth and *M* = 4.00, SD = 2.80 for the seventh, final day. In some entries all emotions belonged to the family of one of the basic emotions, for example “anxiety, fear, disquiet, apprehension,” “indignation, annoyance, rage.” However, in many entries emotions varied in terms of complexity and functions: “anger, sadness, regret” “hopelessness, helplessness, anger, irritability, disappointment, discouragement, regret.”

After aggregating the results, we checked the descriptive statistics; namely, we computed mean and standard deviations for each day to ensure that our participants were reporting intense emotional states. The results were: *M* = 6.21, *SD* = 1.87 for the first day, *M* = 6.54, *SD* = 2.10 for the second, *M* = 6.78, *SD* = 1.81 for third, *M* = 6.78, *SD* = 1.96 for fourth, *M* = 7.19, *SD* = 1.91 for fifth, *M* = 6.88, *SD* = 1.91 for sixth, and *M* = 7.13, *SD* = 1.70 for the seventh. After that we proceeded to analyzing the frequency of appearance for each strategy. It seemed that the most frequently employed strategy was acceptance (present in 44% of diaries entries), then suppression (41%), rumination (39%), reappraisal (36%), and finally, distraction (28%). We checked for the relationships between the intensity of emotions (as a sum of intensities for basic and complex emotional categories along the repeated measurements) and the application of five ERS (rumination, reappraisal, acceptance, distraction, and suppression). We have created separate multilevel linear regression models for each of those strategies (treated as dependent variables). We introduced the emotions intensity (the overall mean of the intensity for all emotions: basic and complex ones; the raw results were cluster-mean centered) and demographic variables (gender and age) as predictors. The application of the particular ERS was treated as dichotomic variables (if the participant has marked that they applied the strategy at least once, we coded this as applied strategy; if not, we coded it as zero) with the exception of distraction strategy (which was put in the model as a continuous variable with the sum of times that participant marked this strategy as present); distraction was, as we have already mentioned, the least used strategy, and for that reason we needed to create a continuous variable as the dichotomic one was too homogeneous, very rarely assuming other values than 0). Furthermore, the emotions intensity was a repeated measurement; thus we nested those results (as a level one variable in the multilevel model) in people subject-wise (level two in the model). In Table 1 we show the results of those five models.

**TABLE 1:** Parameters of the models for five emotion regulation strategies.

Effect					
**Dependent variable**	**Rumination**	**Reappraisal**	**Acceptance**	**Distraction**	**Suppression**
**Fixed effects**					
Intercept	−0.20	−0.78	−0.46	0.43***	−0.41
Intensity of emotions	0.28*** (0.08)	−0.27*** (0.08)	0.07 (0.07)	−0.03 (0.02)	−0.12 (0.06)
Gender	−0.31 (0.29)	0.21 (0.29)	0.96*** (0.31)	0.09 (0.08)	−0.17 (0.24)
Age	−0.005 (0.01)	0.001 (0.01)	−0.002 (0.01)	−0.003 (0.003)	0.002 (0.009)
**Random effects**					
Variance components					
	0.65 (SD = 0.80)	0.69 (SD = 0.83)	0.25 (SD = 0.50)	0.20 (SD = 0.59)	0.25 (SD = 0.50)

For each of the predictors, we report the standardized β coefficients, as well as standard error in the parentheses. All *p*-values are two-tailed and marked with asterisks: ***p < 0.001.

All of the models have good fit statistics – we analyzed the Akaike Information Criterion [*AIC*; (Lorah, 2018)]: for the rumination (*AIC* = 698.70), reappraisal (*AIC* = 681.30), acceptance (*AIC* = 705.50), distraction (*AIC* = 1032.11), and suppression (*AIC* = 732.10). Furthermore, the models of rumination and reappraisal showed accordingly that 15 and 16% of the variance in applying the respective strategy were explained by the inter-personal differences, while the rest was explained by the intra-personal (for the rumination: *ICC* = 0.15; for the reappraisal: *ICC* = 0.16). The intensity of emotions was a statistically significant predictor for rumination (positive predictor) and reappraisal (negative predictor). Furthermore, gender was a significant positive predictor for the acceptance strategy (with man coded as 1).

### 3.2. Intensity of emotions and number of applied strategies

In a similar manner to the previous analyses, we applied the multilevel linear regression to check whether the intensity of experienced emotion is related to the number of the applied ERS. We put (see Table 2) the number of applied strategies in the model (as the sum of all strategies selected by participants) (computed in an identical way as previously and once again cluster-mean centered), gender, and age as predictors.

**TABLE 2:** Parameters of the multilevel regression model explaining the variability of the number of employed emotion regulation strategies.

Effect	
**Dependent variable**	**Number of employed ERS**
**Fixed effects**	
Intercept	2.48*** (0.43)
Intensity of emotions	0.004 (0.04)
Gender	0.61 (0.30)*
Age	−0.005 (0.01)
**Random effects**	
Variance components	
	1.13

For each of the predictors, we report the standardized β coefficients, as well as standard error in the parentheses. All *p*-values are two-tailed and marked with asterisks: *p < 0.05, ***p < 0.001.

Whilst the present model had the fit parameters worse than the previous ones (*AIC* = 1899.29), it was still acceptable, and it explained 48% of the variance as interindividual differences, and 52% as inter-personal variability (*ICC* = 0.48). The only significant predictor was gender (with man coded as 1), showing that men were using statistically 0.60 strategy more than women.

## 4. Discussion

The aim of the study was to analyze the relationship between one of the key emotional factors, namely the intensity, and the choice of emotion regulation strategies used in daily life. We focused on five strategies: distraction, rumination, reappraisal, suppression and acceptance, concerning the successive stages of emotion regulation (Gross, 2015).

The analyses which we conducted partially confirmed the hypotheses. First of all, the intensity of all emotions (both basic and complex) was a significant predictor for applying the strategies of rumination and reappraisal (Table 1). As we anticipated, the nature of this predictor was far different in those two models. In the rumination model, intensity was a positive predictor; therefore, the more intense the emotions were, the more probable it was to employ the rumination strategy. The intensity [and thus the arousal which is correlated with it; (Russell, 1980)] was, in a way, a risk factor for rumination. Studies on the etiology of depression indicate that one of the main factors of the rumination occurrence are stressful life events and emotional reactivity (Clark et al., 1994; Saveanu and Nemeroff, 2012). Ruminating thoughts arise in the aftermath of traumatic and stressful events that involve intense emotions (Nolen-Hoeksema and Morrow, 1991; Michl et al., 2013). Similarly, strong negative experience is a predictor of ruminating about other aspects of life (Morrow and Nolen-Hoeksema, 1990; Nolen-Hoeksema and Morrow, 1991). Furthermore, childhood abuse is associated with greater emotional reactivity and depression problems in adult life (Mullen et al., 1996). Neuroimaging studies indicate increased activity of the bottom-up system (especially in amygdala, hippocampus, rACC) during habitual rumination combined with reduced top-down cognitive control (Disner et al., 2011; Mandell et al., 2014). This indicates that strong emotional arousal (caused by extremely stressful or perceived as one stimuli) may imply the automatic use of rumination.

Nevertheless, this effect was exactly opposite for the reappraisal strategy, for which the intensity was a negative predictor; thus, the higher the intensity, the lower probability of employing this particular strategy. Perhaps this effect might also be explained by the arousing property of the intensity of emotions, which may block the cognitive strategy such as reappraisal. Both reappraisal and rumination are related to the cognitive interpretation of the event. However, unlike passive and automatic rumination, reappraisal involves cognitive effort to change the perception of the situation. Furthermore, reappraisal is a complex process, the activation of which mainly involves prefrontal cortex areas (PFC) (Ochsner and Gross, 2005; Buhle et al., 2013) and attentional top-down control (Ligeza et al., 2016). According to attention and information processing theories, people have limited cognitive capacity to execute mental operations (e.g., Rohrer et al., 1998; Baumeister et al., 2001). Intensity of arousal affects the response systems which formulate an emotional response (Bradley et al., 2001) and influence regulatory capabilities. Since reappraisal requires greater availability of cognitive resources, its implementation may be thwarted by strong arousal.

We did not obtain significant results between emotion intensity and regulation choices of acceptance, distraction and suppression. However, in the case of suppression the intensity of emotions was a negative predictor on the border of the statistical trend (*p* < 0.07). The obtained results may indicate that the intensity of emotions does not have such a big impact on the choice of these strategies in complex situations, in which other contextual and motivational factors are important.

Another significant predictor in this part of our results was gender. As we see, it is a positive predictor for the acceptance strategy; it means that men are more likely to choose this strategy than women in various stressful situations. This is an interesting result that requires further exploration. In Flynn et al.’s (2010) study women more often reported a non-accepting approach to their emotions than men did. They also had more depressive symptoms (Flynn et al., 2010). In the aspect of attitude toward emotions, perhaps acceptance is the opposite of rumination, which is associated with negative evaluation of one’s own affective experience.

Contrary to what we expected, we did not obtain significant results for the intensity of emotions. The result might be the effect of the employed procedure. Selected situations were characterized by the strongest unpleasant emotions of the day. The variance in the intensity of emotions could therefore result from situational factors (how stressful were the events of the day) and individual differences in emotional reactivity. Thus, the number of selected strategies was more related to situational and individual factors than to the intensity of emotions.

We also checked whether the gender and age may explain the variability in the number of different strategies employed (Table 2). The effect of age turned out to be insignificant, which may be due to the fact that the study involved mature people in the age range 30–50. Studies on age cohorts indicate the greatest differences in ERS between adolescents, mature and older people (Zimmermann and Iwanski, 2014). However, we observed an interesting effect for the gender, being once again a significant predictor. It shows that men are more likely to use a higher number of strategies than women. This result stands in contrast to previous studies. Women were reported to use a wider variety of both adaptive and maladaptive regulation strategies than men (Thoits, 1991; Tamres et al., 2002; Aldao and Nolen-Hoeksema, 2010). However, they were also reported to experience negative emotions more often and more intensely (Fujita et al., 1991). This may be connected with greater emotional reactivity (Domes et al., 2010) and the fact of appraising stressors as more severe (Tamres et al., 2002). In our study, participants reported using the ER strategy in relation to the situation that aroused their strongest unpleasant emotions during the day. Perhaps men do not have the motivation to attach emotional meaning to a number of events and relations as much as women do (Ickes et al., 2000; Klein and Hodges, 2001). Nevertheless, in a situation perceived as stressful and worthy of emotional involvement they use even more strategies to regulate their emotions.

The use of the daily diary method in measuring emotion regulation choices has its advantages and limitations. The most important of them are the subjectivity of the assessment and possible distortions of memories (Levine, 1997). However, we focused on the participants’ individual assessment of their emotional experience, in particular its intensity. We assumed it is the people’s individual perception and interpretation of the event that controls regulation choices to the highest degree. Experiences reported by the respondents were diverse. They concerned work, family, and usually happened in an interpersonal context. The experience-sampling procedures are dependent on naturally occurring events and experiences, and do not allow for a standardized procedure to control all situational variables (Christensen et al., 2003). Nevertheless, the concern of this research was to look at making regulatory decisions in personally relevant and complex everyday situations. In standardized laboratory studies the stimuli are perceived differently by the participants and the research lacks everyday life realism.

What distinguishes the method used in our study is the ability to freely name the emotional experience during an unpleasant situation. This gives us additional information proving that the emotional attributions experienced in everyday life are different and tend to vary in function and complexity. Although the emotions experienced in response to a difficult situation were varied, the intensity of the emotional experience was an important factor determining the choice of a regulation strategy. It indicates that this emotional dimension is a basic and determining aspect in people’s regulatory capabilities.

In the study, the average arousal of the emotions reported by the participants usually concerned several negative complex and basic emotions. Emotions such as fear or anger may require other ways of regulation. It is worth looking at in further research how the intensity of discrete emotions, both basic and complex ones, may influence the choice of regulation strategies. Perhaps this specificity of the regulation strategy could be the reason behind the fact that in our study the variance explained by the models (the ICC values) was rather low, around 15% (Koo and Li, 2016); perhaps controlling for the category of emotions (either discrete or dimensional ones, e.g., controlling for valence) could increase the variance explained by the intensity of specific emotions in applying the particular strategy. This result also shows that the way of reacting among healthy adults is determined by situational factors (a type of experienced difficulties) and the choice of an emotional regulation strategy is flexibly adapted to them.

The obtained results are consistent with the theory and indicate the importance of the influence of the intensity of emotional arousal on the everyday choice of regulation strategies. Data obtained using the diary method may be a valid criterion for the results acquired in other research paradigms. They not only broaden the already existing knowledge about how we manage our emotions, but may also find an application in all kinds of psychological interventions (e.g., psychotherapy, anger management therapies), allowing to better understand the choice of emotion regulation strategies.

## Data availability statement

The raw data supporting the conclusions of this article will be made available by the authors, without undue reservation.

## Ethics statement

The studies involving humans were approved by the Ethics Committee of the Faculty of Psychology of the University of Warsaw protocol code 19/3/2019 date of approval 19 March 2019. The participants provided their written informed consent to participate in this study.

## Author contributions

MK and AS: conceptualization, methodology, validation, supervision, and funding acquisition. MK: software, investigation, resources, and project administration. AW and MK: formal analysis. All authors contributed to the writing–original draft preparation, writing–review and editing, and visualization and read and agreed to the published version of the manuscript.
