# Supplementary material for: Emotional regulation strategies in daily life: the intensity of emotions and regulation choice
Source: Front Psychol. 2023 Aug 14;14:1218694. doi: 10.3389/fpsyg.2023.1218694 (PMC10460911; doi:10.3389/fpsyg.2023.1218694)
Supplement: Supplementary file 1 [file Data_Sheet_1.docx]

Supplementary Material

Emotional regulation strategies in daily life: the intensity of emotions and regulation choice

Magdalena Kozubal*, Anna Szuster, Adrianna Wielgopolan

*** Correspondence:** Corresponding Author: [magdalena.kozubal@psych.uw.edu.pl](mailto:magdalena.kozubal@psych.uw.edu.pl)

**A**ppendix 1

Re-1 => Pomyślałem, że to mało ważne

Re-2 => Pomyślałem o tym co dobrego może mi przynieść ta sytuacja, potraktowałem ją jako okazję do nauki

Re-3 => Pomyślałem o tym jak odbiera tę sytuacje inna osoba

S-1 => Starałem się nie odczuwać tych uczuć, które się pojawiły

S-2 => Mimo, że odczuwałem emocje starałem się ich po sobie nie pokazywać

S-3 => Starałem się nie myśleć o tej sytuacji

A-1 => Przyjąłem tę sytuację taką jaka ona jest

A-2 => Akceptowałem uczucia jakie ta sytuacja we mnie wzbudziła

A-3 => Akceptowałem siebie w tej sytuacji z uczuciami, które we mnie wzbudziła

D-1 => Starałem się pomyśleć o czymś przyjemnym, pozytywnym

D-2 => Zająłem się czymś innym co nie było związane z tą sytuacją

D-3 => Postanowiłem pomyśleć o czymś innym niezwiązanym z tą sytuacją

Ru-1 => Zagłębiałem się we własne myśli i odczucia związane z tą sytuacją

Ru-2 => Koncentrowałem się na uczuciach, które wywołała we mnie ta sytuacja

Ru-3 => Myślałem o tym jakie są przyczyny tej sytuacji i jakie będzie miała skutki dla mnie

English translation

Re-1 => I thought it was not important

Re-2 => I thought of how I could benefit from this situation, I treated it as a lesson to learn

Re-3 => I thought of how this situation can be perceived by a different person

S-1 => I tried not to take the feelings which appeared

S-2 => Even though I felt the emotions, I tried not to show them

S-3 => I tried not to think about this situation

A-1 => I accepted this situation the way it was

A-2 => I accepted the feelings which were evoked by this situation

A-3 => I accepted myself in this situation with all the feelings it evoked inside of me

D-1 => I tried to think about something nice, positive

D-2 => I started doing a different thing which was not connected with this situation

D-3 => I decided to think about something different which was not connected with this situation

Ru-0 => I got deeper into my own thoughts and feelings connected with this situation

Ru-1 => I concentrated on the feelings evoked by this situation

Ru-2 => I wondered what were the causes of this situation and what consequences it would have for me

**A**ppendix 2

| radość  rozbawienie  szczęście  entuzjazm  ekscytacja  nadzieja  ufność  optymizm  duma  satysfakcja  spełnienie  zadowolenie  szacunek  podziw  zachwyt  respekt  miłość  uwielbienie  sympatia | akceptacja  ulga  spokój  błogość  zmieszanie  niepewność  zagubienie  przytłoczenie  zaciekawienie  zainteresowanie  oczekiwanie  zdziwienie  zaskoczenie  gniew  rozdrażnienie  oburzenie  złość  zniecierpliwienie  wściekłość | nienawiść  pogarda  wstręt  obrzydzenie  odraza  smutek  przygnębienie  żal  rozpacz  rozczarowanie  zwątpienie  bezradność  bezsilność  beznadzieja  strach  lęk  groza  niepokój  obawa | poddenerwowanie  przerażenie  panika  skrucha  poczucie winy  wstyd  zakłopotanie  zażenowanie  rozczarowanie  zawód  zniechęcenie  zazdrość  osamotnienie  odrzucenie  zranienie  poczucie krzywdy |
| --- | --- | --- | --- |

**E**nglish translation

| joy  amusement  happiness  enthusiasm  excitement  hope  trust  optimism  pride  fulfillment  satisfaction  respect  admiration  delight  love  adoration  liking | acceptance  relief  composure  bliss  confusion  uncertainty  lostness  overwhelm  interest  expectancy  surprise  anger  irritability  indignation  anger  impatience  rage | hate  contempt  disgust  sadness  depression  regret  desperation  disappointment  doubt  helplessness  hopelessness  fear  terror  anxiety  apprehension | nervousness  horror  panic  repentance  guilt  shame  embarrassment  disappointment  discouragement  jealousy  solitude  rejection  feeling hurt  sense of wrong |
| --- | --- | --- | --- |
